# Imaging and Quantitative Analysis of the Interstitial Space in the Caudate Nucleus in a Rotenone-Induced Rat Model of Parkinson’s Disease Using Tracer-based MRI

**DOI:** 10.14336/AD.2016.0625

**Published:** 2017-02-01

**Authors:** Deyong Lv, Jingbo Li, Hongfu Li, Yu Fu, Wei Wang

**Affiliations:** ^1^Department of Radiology, Peking University Third Hospital, Beijing 100191, China; ^2^Beijing Key Laboratory of Magnetic Resonance Imaging Device and Technique, Beijing 100191, China; ^3^Department of Radiology, Dongying People’s Hospital of Shandong, Shandong, 257091, China; ^4^Department of Ultrasound, Dongying People’s Hospital of Shandong, Shandong, 257091, China; ^5^Department of Neurology, Peking University Third Hospital, Beijing 100191, China

**Keywords:** Magnetic Resonance, Parkinson’s disease, Interstitial Space, Brain, Diffusion

## Abstract

Parkinson’s disease (PD) is characterized by pathological changes within several deep structures of the brain, including the substantia nigra and caudate nucleus. However, changes in interstitial fluid (ISF) flow and the microstructure of the interstitial space (ISS) in the caudate nucleus in PD have not been reported. In this study, we used tracer-based magnetic resonance imaging (MRI) to quantitatively investigate the alterations in ISS and visualize ISF flow in the caudate nucleus in a rotenone-induced rat model of PD treated with and without madopar. In the rotenone-induced rat model, the ISF flow was slowed and the tortuosity of the ISS was significantly decreased. Administration of madopar partially prevented these changes of ISS and ISF. Therefore, our data suggest that tracer-based MRI can be used to monitor the parameters related to ISF flow and ISS microstructure. It is a promising technique to investigate the microstructure and functional changes in the deep brain regions of PD.

The interstitial space (ISS) within the brain provides an immediate accommodation space for neural cells and contributes to the physiological and functional homeostasis of the brain, where the flow of interstitial fluid (ISF) is important for nutrient supply, waste removal and intercellular communication [1]. Parkinson’s disease (PD) is the primary neurodegenerative disease of the basal ganglia and is characterized by neurotransmitter alterations in the caudate nucleus and selective loss of dopaminergic neurons in the substantia nigra [2-4]. Studies have demonstrated microstructural changes in the ISS of PD [5, 6]. However, changes in ISF flow within the caudate nucleus of PD have not been reported. The caudate nucleus is vulnerable to the effects of PD and is the target region of several promising therapeutic strategies [7, 8]. Therefore, investigating the changes in ISS and ISF flow in the caudate nucleus is necessary to comprehensively understand the mechanisms underlying pathogenesis of PD and optimize the efficacy of therapeutic strategies. The width of the ISS ranges from 38-64 nm, which, therefore, is challenging to image *in vivo* [9]. To our knowledge, the tracer-based MRI technique is a unique *in vivo* method that can measure both the microstructure of ISS and ISF flow in deep brain regions [10, 11]. In tracer-based MRI, the tracer is introduced into the ISS of the target region, and the radiofrequency signal is assessed using MRI. The tracer concentration is calculated by the signal intensity of the images, and the parameters of the microstructure and flow can be calculated according to the diffusion equation. In the present study, we investigated the changes of ISS in the caudate nucleus in a rotenone-induced model of PD, with or without madopar treatment, using tracer-based MRI.

## MATERIALS AND METHODS

### Rotenone-induced injury, madopar treatment rat model and behavioral testing

Thirty 8-week-old male Sprague-Dawley rats (280 g-320 g) were randomly divided into three groups (n=10): (a) rotenone-induced PD group that received daily subcutaneous injections with rotenone solution (1.5 mg/kg/day) [12], (b) madopar-treated group that received both rotenone injections and intragastric administration with madopar (50 mg/kg/day) and (c) sham group that received subcutaneous injections with saline. All rats were administrated the above mentioned treatment for 4 weeks. Motor impairment in the rotenone-induced group was verified using the hanging-wire and inclined plane tests [13, 14]. All animal experimental procedures were reviewed and approved by the Peking University Institutional Animal Care and Use Committee and the Peking University Committee on Animal Care (No. LA2012-016).

**Figure 1.**
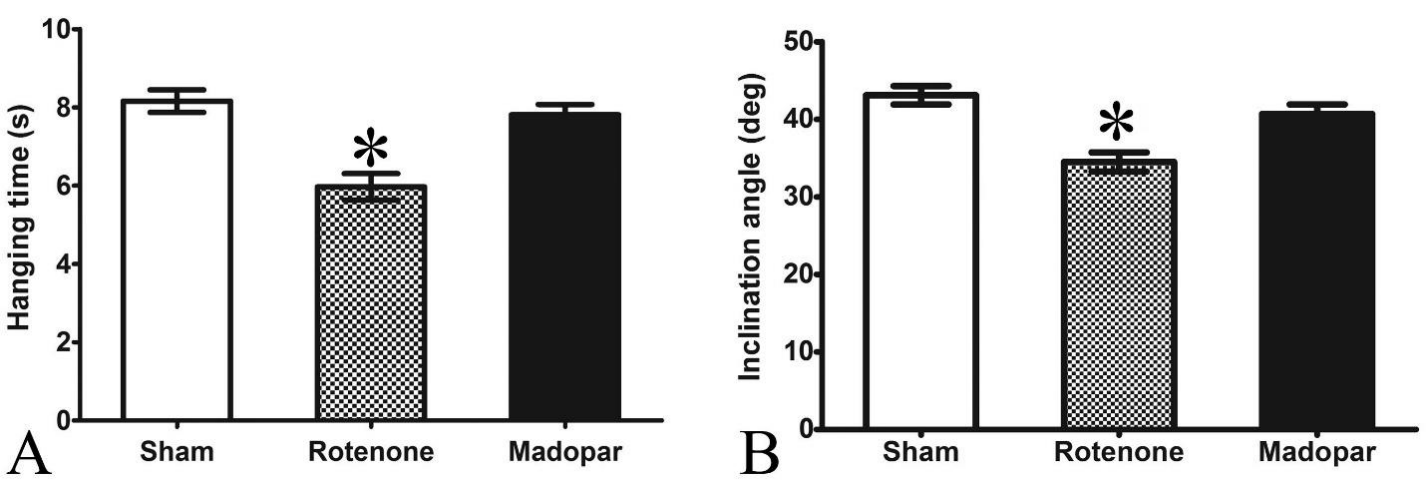
**Behavioral changes after treatment**. (**A**) The average hanging time in the hanging-wire test four weeks after rotenone-induced damage and madopar treatment. (**B**) The average inclination angle in the inclined plane test four weeks after rotenone-induced damage and madopar treatment. Both the hanging time and the inclination angle were significantly decreased in the rotenone-induced group compared to the other two groups. Data are the mean ± SEM (n = 10). One-way ANOVA and SNK tests were performed, * represents *P* < 0.05.

### Tracer-based MRI technique

The tracer-based MRI technique was perfomed according to the propotocol previously described by Han [10, 15]. Rats were anesthetized by a combination of pentobarbital sodium, ethanol, chloral hydrate, magnesium sulfate and propylene glycol (3 ml/kg) via intraperitoneal injection. Anesthesia was subsequently maintained with additional injections over the course of the experiment (approximately 0.7 ml/kg/h). The rats were fixed in a stereotactic apparatus, an incision was made in the scalp along the sagittal suture, then the bregma was exposed. A small trephine hole in the skull bone was made according to the stereotactic coordinates of the caudate nucleus (bregma: +1.0 mm, lateral: 3.5 mm, vertical: 5.0 mm) [16]. 10 mmol/L of gadolinium-diethylene triamine pentacetic acid (Gd-DTPA) in 2 μl was injected into the caudate nucleus of each rat by a syringe pump (rate: 0.2 μL/min). After the injection, the needle was left in place for an additional 5 min and then slowly withdrawn. MR scanning was operated in a 3.0-Tesla MRI system (Magnetom Trio; Siemens Medical Solutions, Germany) with an eight-channel wrist coil using T_1_ 3D MPRAGE sequences. MR scanning was performed sequentially pre-injection and post-injection (0.5, 1, 1.5, 2, 3, 4, 5, 6, 7 and 8 h). The scanning parameters were as follows: repetition time = 1500 ms, echo time = 3.7 ms, flip angle = 12°, inversion time = 900 ms, field of view=267 mm, voxel = 0.5 × 0.5 × 0.5 mm^3^, matrix = 512 × 512 and acquisition time = 290 s. The axial images of the same anatomical site from different time points were imported into the processing workstation for quantitative analysis. The increment in the signal intensity of all of the pixels in the image was recorded after registration and subtraction procession using Matlab (MathWorks, Inc., Natick, MA) and were converted to the concentration of Gd-DTPA [17]. We extracted the parameters related to the microstructure (effective diffusion parameter D* and tortuosity λ) and clearance of Gd-DTPA (clearance rate constant *k*’ and half-life t_1/2_). D* represents the diffusion ability of substances in the ISS and is less than the free diffusion parameter (D) due to the diffusion barrier in the ISS. λ can be calculated by the equitation λ= $\sqrt[{2}]{\text{D}/\text{D}*}$ and is related to the microstructure of the ISS.*k*’refers to nonspecific uptake and represents the loss of Gd-DTPA due to cell metabolism. t_1/2_ is the amount of time needed for a reactant concentration to decrease by half compared to the initial concentration of Gd-DTPA. Almost all Gd-DTPA is cleared from the ISS through the paravascular spaces surrounding large draining veins and is drained into the cerebrospinal fluid and the lymphatic system [18]. Therefore, t_1/2_ is primarily related to the diffusion and bulk flow of ISF.

### Statistical analysis

One-way analysis of variance (ANOVA) followed by Student-Newman-Keuls (SNK) test was performed for comparisons among the groups with SPSS 18.0. Statistical significance was set a priori at 0.05.

**Figure 2.**
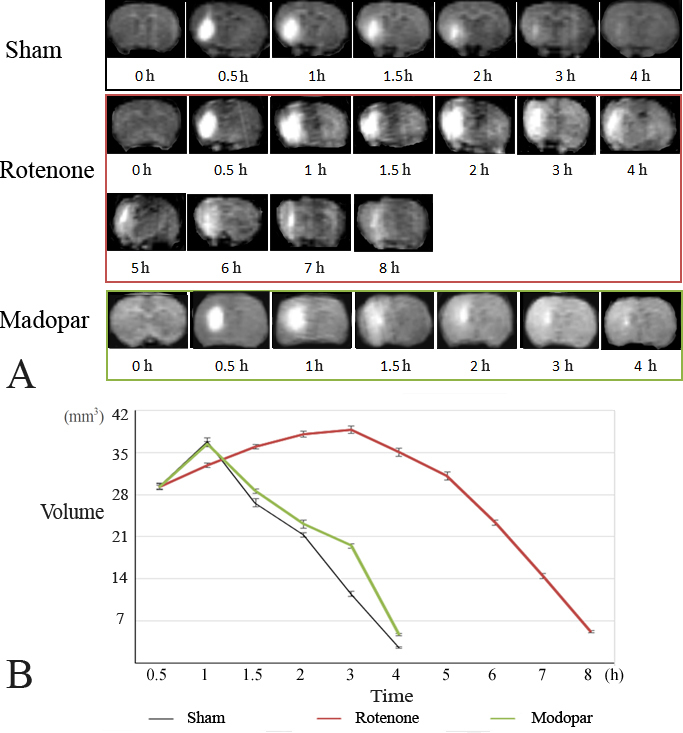
**Axial views of MRI and spatiotemporal distribution pattern of Gd-DTPA after injection into caudate nucleus**. (**A**) Axial MR images of the spreading tracer in the ISS at different time points after the introduction of Gd-DTPA. Gd-DTPA introduced into the ISS can lighten the water molecules and increase the signal intensity in the spreading regions. The transportation and clearance of Gd-DTPA can be demonstrated using a series of MR images. (**B**) Line chart of the distribution region at the different time points. The volume of each pixel in the MRI is 0.5×0.5×0.5 mm^3^, and the volume amount of the “lightened” regions in each image for each time point was calculated. The maximum spreading region was not significantly different among the three groups. Data are the mean ± SEM (n=10). SNK test was performed.

## RESULTS

Compared with the sham group (8.16 ± 0.91 s) and the madopar-treated group (7.81 ± 0.85 s), the average hanging time in the rotenone-induced model group was significantly decreased (5.97 ± 1.07 s) in the hanging-wire test (*P* < 0.05). In addition, compared with the sham group (43.1 ± 3.8°) and the madopar-treated group (40.7 ± 3.8°), the average inclination angle in the inclined plane test was significantly decreased in the rotenone-induced group (34.5 ± 3.9°, *P* < 0.05). There were no significant differences between sham group and the madopar-treated group in either the hanging-wire or the inclined plane test (Fig. 1).

In the sham and madopar-treated groups, a maximum spreading region was reached at 1 and 4 h after the introduction of Gd-DTPA, and the Gd-DTPA had almost completely disappeared. In the rotenone-induced group, a maximum spreading region was observed 3 h after the introduction of Gd-DTPA and the tracer was eliminated 8 h later. The maximum spreading region was not significantly different among the three groups (*P* > 0.05) (Fig. 2)

In this study, we measured parameters related to the microstructure of the ISS (D *, λ) and the clearance of Gd-DTPA (*k* ’, t_1/2_). Compared with the sham group, D* was significantly increased (5.828 ± 0.727 vs 2.770 ± 0.506 × 10^−4^ mm^2^/s, *P* < 0.05), and λ was significantly decreased (0.916 ± 0.209 vs 1.560 ± 0.320, *P*< 0.05) in the rotenone-induced group. However, compared with the sham group, the amplitude of variation was smaller in the madopar-treated group (D* = 3.645 ± 0.451×10^−4^ mm^2^/s, λ = 1.157 ± 0.128, *P* > 0.05). Moreover, a significant difference was found between the rotenone-induced group and the madopar-treated group (*P* < 0.05).

**Figure 3.**
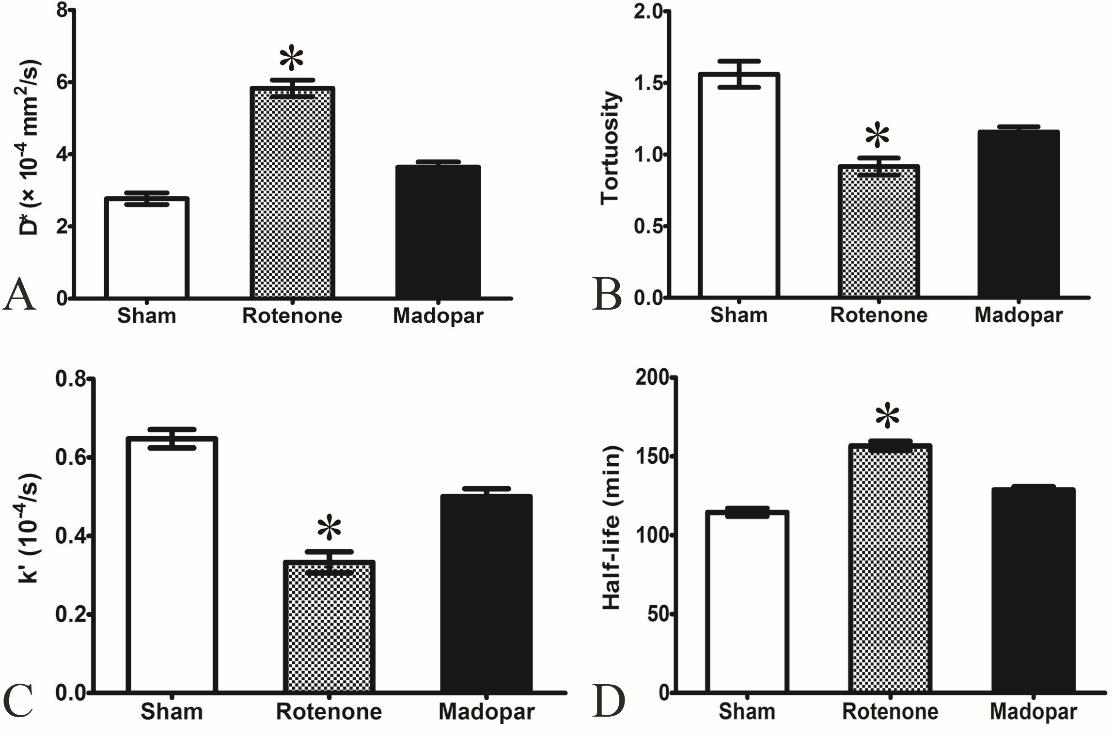
Alterations in the microstructure parameters (D*, λ) and the clearance of Gd-DTPA (*k*’, t_½_) in the caudate nucleus after rotenone-induced damage and madopar treatment. (**A**) Compared to the sham group, the effective diffusion parameter(D*) was significantly increased in the rotenone-induced group. No differences were observed between the sham group and the madopar-treated group. (**B**) Compared to the sham group, tortuosity (λ) was significantly decreased in the rotenone-induced group. No differences were observed between the sham group and the madopar-treated group. (**C**) Compared to the sham group, the clearance rate constant (*k*’) was significantly decreased in the rotenone-induced group. No differences were observed between the sham group and the madopar-treated group. (**D**) Compared to the sham group, half-life (t_½_) was significantly prolonged in the rotenone-induced group. No differences were observed between the sham group and the madopar-treated group. Data are the mean ± SEM (n=10); SNK test was performed. * represents *P*<0.05

The results showed that the *k* ’ in the rotenone-induced group was significantly decreased (*k* ’ = 0.333 ± 0.093 × 10^−4^/s), and t_½_ was significantly prolonged (t_½_ = 156.6 ± 10.2 min) in comparison with the sham group (*k* ’ = 0.648 ± 0.082 × 10^−4^/s, t_½_ = 114.6 ± 8.7 min) and the madopar-treated group (*k* ’ = 0.500 ± 0.070 × 10^−4^/s, t_½_ = 128.8 ± 7.1 min) (Fig. 3).

## DISCUSSION

In this study, using tracer-based MRI allowed us for the first time to visualize the changes in ISF flow in the caudate nucleus in the rotenone-induced rat model of PD. We demonstrated that the ISF flow slowed and that the tortuosity of the ISS significantly decreased in the rat model. Madopar administration partially prevented these changes of ISS and ISF.

Tracer-based MRI was used in the current study, because this technique has the ability to image dynamic ISF flow in the brain from a global view. To date, three techniques have been developed to investigate the ISS *in vivo*, including real-time iontophoresis (RTI), integrative optical imaging (IOI) and tracer-based MRI. RTI can accurately evaluate the diffusion parameters of the ISS across a distance of approximately 100-200 µm, but it cannot visualize ISF flow. Similar to tracer-based MRI, IOI can visualize ISF flow using a fluorescent tracer to calculate the parameters. However, IOI is limited to superficial (200 µm) brain regions and cannot be used to investigate the ISS of deep brain regions, where the structure produces too much light scattering [9, 19]. Tracer-based MRI utilizes a magnetic tracer (Gd-DTPA), which can shorten the spin-lattice relaxation time of hydrogen nuclei in water molecules within an effective distance of 2.5 angstroms. This extracellular probe increases the signal intensity of MRI.

According to our findings, the microstructure changes in the ISS measured using tracer-based MRI are consistent with previous reports using RTI [6]. Furthermore, we have verified that there are biophysical changes in ISS in the deep brain nuclei in PD. Importantly, our recent report using a 6-hydroxydopamine-induced rat model of PD demonstrated that there was decreased tortuosity and a reduced clearance rate in the ISS of substantia nigra [5]. The changes were partially mediated by dopaminergic neuron loss and reactive astrogliosis. In the current study, we demonstrated similar changes in the ISS of the caudate nucleus using a rotenone-induced rat model of PD. Whereas, unlike the pathological changes in the substantia nigra, no extensive cell loss or reactive astrogliosis have been observed in the caudate nucleus in PD [13, 20]. We hypothesize that the changes in the ISS in the caudate nucleus may be caused by the degeneration of dopaminergic axons and the up-regulation of spontaneous neuronal discharge activity [13, 21]. The boundary structure of the ISS, which is made of the cell membrane and extracellular matrix, prevents the diffusion of substances within the ISS. Moreover, unpublished work from our lab showed that myelin fibers act as a barrier to ISF flow. Axon degeneration can alter brain structures and influence diffusion parameters. Additionally, in a recent study, we demonstrated that functional neuronal discharge activity in normal rats can slow ISF flow and reduce clearance of Gd-DTPA [22].

In future, application of this method will provide new insight into the mechanisms of PD. For example, this technique may be used to answer the following question: “Where PD begins at the cellular level, the neuron soma in the substantia nigra or the axonal terminal in the striatum?”. Moreover, current treatment of PD is not clinically satisfied, and pharmaceutical drugs offer only time-limited symptomatic relief and become less effective as the disease progresses [23]. Emerging ISS administrations that involve gene therapies and cell transplantation have shown promise in the treatment of PD [24, 25]. ISS administration provides a number of advantages over conventional administration, including bypass of the blood brain barrier, minor systemic toxicity and enhanced efficacy, etc [26]. Real-time monitoring and quantitative analysis of the changes in the ISS are essential to both optimizing these treatment strategies and evaluating their efficacy. Our results validated the practicality and sensitivity of tracer-based MRI to monitor the changes in the ISS of the deep brain nuclei of treated or untreated PD.

It is important to note that we report only a novel application of tracer-based MRI in this study and future pathological or histological studies require investigations.
